# The effect of blue light filtering lenses on speed perception

**DOI:** 10.1038/s41598-021-96941-0

**Published:** 2021-09-02

**Authors:** Adiba Ali, Maitreyee Roy, Hind Saeed Alzahrani, Sieu K. Khuu

**Affiliations:** 1 School of Optometry and Vision Science, University of New South Wales Sydney, Sydney, NSW 2052 Australia; 2 Department of Physics, Taif University, Ta’if, Saudi Arabia

**Keywords:** Vision disorders, Patient education

## Abstract

Blue-light filtering lenses (BFLs) are marketed to protect the eyes from blue light that may be hazardous to the visual system. Because BFLs attenuate light, they reduce object contrast, which may impact visual behaviours such as the perception of object speed which reduces with contrast. In the present study, we investigated whether speed perception is affected by BFLs. Using a two-interval forced-choice procedure in conjunction with Method of Constant Stimuli, participants (n = 20) judged whether the perceived speed of a moving test stimulus (1.5–4.5°/s) viewed through a BFL was faster than a reference stimulus (2.75°/s) viewed through a clear lens. This procedure was repeated for 3 different BFL brands and chromatic and achromatic stimuli. Psychometric function fits provided an estimate of the speed at which both test and reference stimuli were matched. We find that the perceived speed of both chromatic and achromatic test stimuli was reduced by 6 to 20% when viewed through BFLs, and lenses that attenuated the most blue-light produced the largest reductions in perceived speed. Our findings indicate that BFLs whilst may reduce exposure to hazardous blue light, have unintended consequences to important visual behaviours such as motion perception.

## Introduction

**Figure 1:**
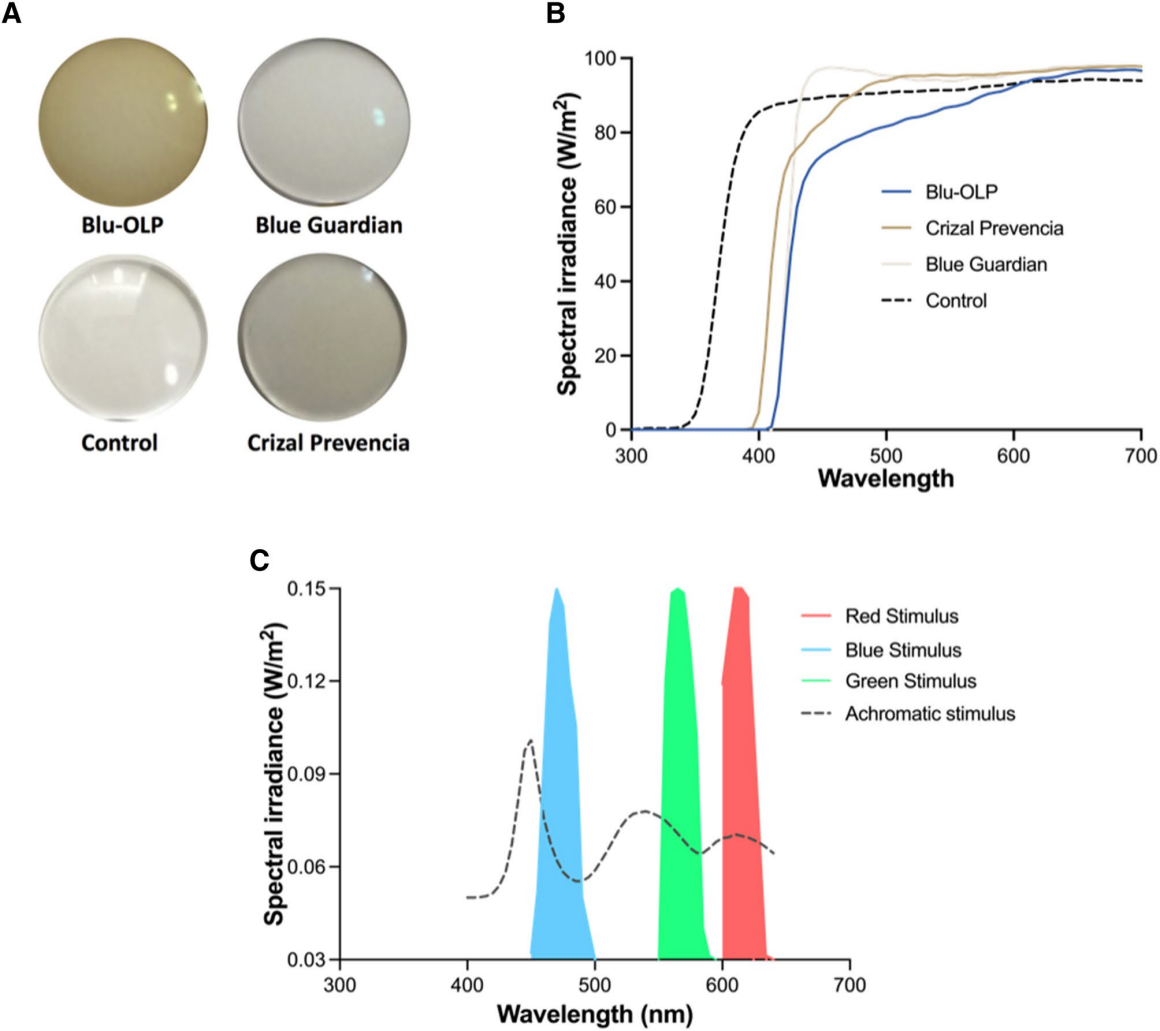
In (**A**), the four different lenses used in the present study to examine their effect on perceived speed. In (**B**), transmittance function of the difference lenses used in the present study is shown. Each function quantifies the degree to which each lens reduces light transmittance over a range of wavelengths. In (**C**), are the spectral power distribution of the 4 stimuli used in the present study is shown.

Blue light filtering lenses (BFLs) selectively reflect or absorb blue light (440–500 nm) by 6–43%^1^, with newer generation BFLs (see Fig. 1A) attenuating light within this range to preserve some of the benefits of blue light, particularly to the circadian rhythm and blue light perception. BFLs are sold commercially with claims to protect the eyes from the harmful effects of blue light, such as improve sleep at night^2^ and protect the retina against Age-Related Macular Degeneration^3^. BFLs have different spectral transmittance characteristics depending on the brand^1^, which allows them to selectively attenuate light at different intensities, particularly at shorter wavelengths. However, selectively filtering blue light may lead to unintended consequences such as alterations to normal visual functions, as has been reported by a number of previous studies^4–7^. Yet, BFLs are produced and sold commercially without the manufacturers or consumers being aware of its effects on visual function^8^.

As BFLs selectively reduce the amount of blue light reaching the eyes, it is expected that there will be an overall reduction in image contrast, particularly if the reflectance/light-emitting properties of the object comprise predominantly short wavelengths^6,7^, whilst leaving other wavelengths unaffected. Theoretically, the properties of BFLs may decrease contrast perception due to the attenuated amount of blue light reaching the S-cones, as complete activation of S-cones influences luminance contrast^9^. Therefore, blue light-filtering intraocular lenses (IOLs) reduce contrast detection, and it is possible that BFLs may also cause a similar effect. Our previous study demonstrated that 7 commercially available BFLs reduced the detection of blue colours by 5–36%^1^, suggesting that they potentially would affect the luminance contrast perception of objects. Indeed, previous studies have shown that the contrast sensitivity function and total error score for participants wearing yellow and orange IOLs were lower than those without, and the effect was more pronounced under mesopic conditions (3 cd/m^2^) than photopic conditions^5–7,10^. In a study by Leung and colleagues, contrast discrimination was not significantly affected by BFLs. It is important to note that their study utilised the MARS Contrast Sensitivity test, which is subjective and used large fixed contrast steps^11^, and therefore may not have sufficient sensitivity to detect a change in contrast detection ability. Indeed, our recent work using computerised stimuli and psychophysical methods that allowed for more precise contrast steps has shown that BFLs can reduce object luminance and colour contrast sensitivity^12^ and affect photostress recovery times^13^, particularly at low contrast levels consistent with the mesopic vision.

**Figure 2:**
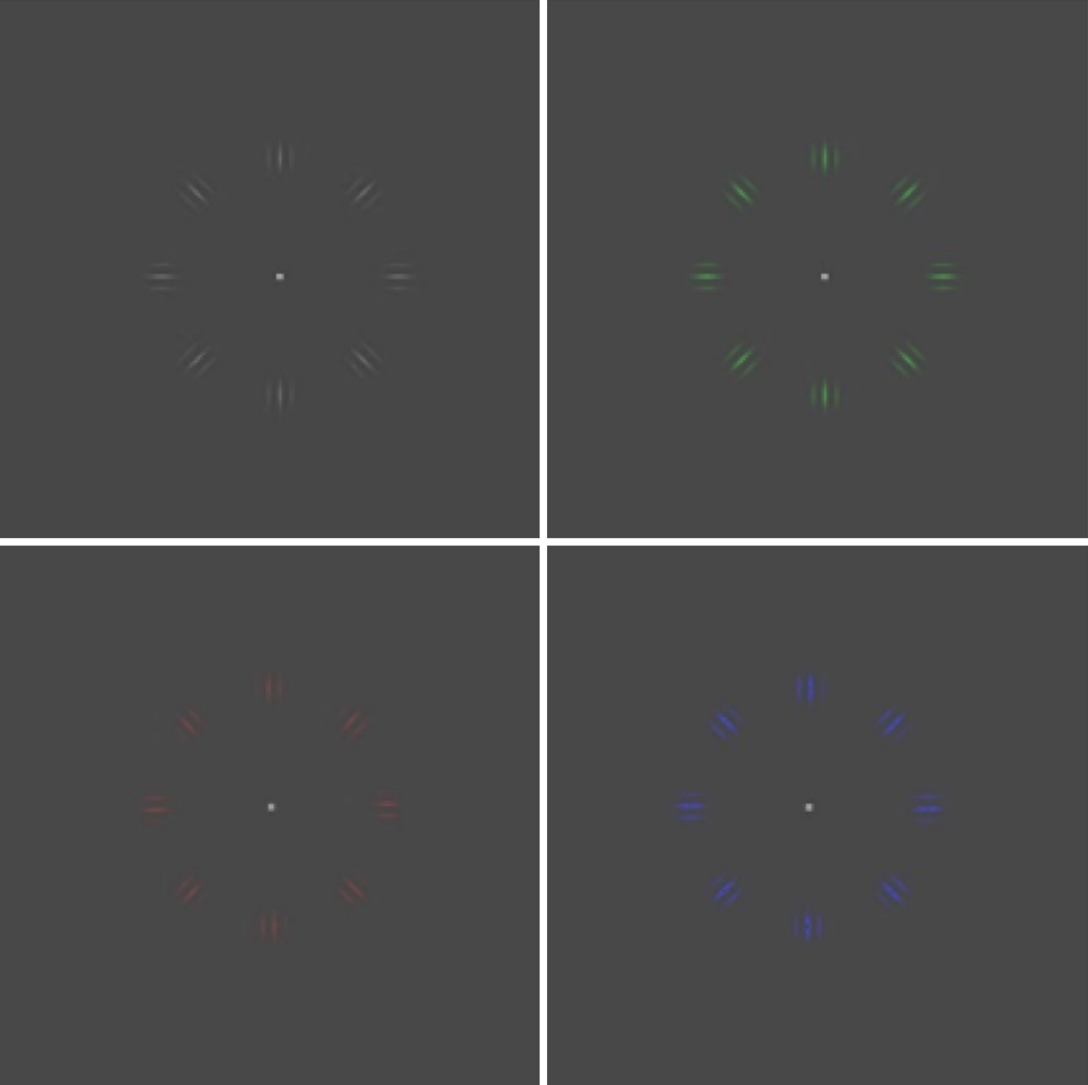
Images of the annulus (comprising of 8 Gabor elements) stimuli used in the present study which were either achromatic, green, red and blue in colour.

Previous studies have well established that a number of visual behaviours are dependent on luminance contrast. For example, a visual behaviour that has been shown to depend on contrast is perceived object speed which is the perception of the rate at which object is moving in a particular direction^14^. In particular, perceived speed is perceptually reduced with luminance contrast, and this effect is greatest at low contrast and slow speeds^14–26^. In a classic study by Snowden et al.^27^, it was demonstrated that the perceived speed of optic flow patterns decreased with reduced contrast when participants used a driving simulator with quasi-natural scenes^27,28^. This caused drivers to ‘speed up’ to match the impression of their travelling speed. Owens et al.^27^ performed a similar study on a rural road and demonstrated that the reduced visibility led to a significant decrease in perceived speed. Theoretically, if BFLs reduce perceived contrast, then perceived speed should also be reduced as a result. Note that BFLs selectively reduce light transmission at short wavelengths, but do not impede the transmission of medium and longer wavelengths (see “Methods” section). Accordingly, this selective filtering may reduce the contrast of a blue object on certain backgrounds such as a neutral gray (as used in the present study, see Figs. 1, 2).

Interestingly, whilst reduction in contrast decreases perceived speed, previous reports^29,30^ has shown that reducing overall scene luminance has the effect of increasing perceived object speed. Under such conditions, the contrast of the object remains the same and both the object and background luminance are similarly attenuated. Reductions in overall scene luminance are known to reduce driving speeds as perceived speed perceptually increases^30^.

Given the noted relation between perceived speed and luminance contrast, and that BFLs attenuate luminance, the present study aimed to establish whether BFLs affect speed perception and if so, quantify this effect. In our study, observers judged the perceived speed of an annulus of Gabor elements simulating rotating motion that could be one of 3 different colours (see Figs. 1C, 2). In a trial, one of these stimuli was viewed through a BFL and compared with another similar stimulus viewed through a single clear control lens. This experimental approach was repeated for 3 different commercially available BFLs; Blu-OLP (GenOp), Crizal Prevencia (Essilor) and Blue Guardian (Opticare). The spectral transmittance characteristics of these lenses (see Fig. 1A) have been previously reported and are different in regard to the extent of light filtration for all wavelengths of light. Blu-OLP and Crizal Prevencia were previously revealed to transmit the least blue light, whereas Blue Guardian possessed one of the highest blue light transmittance profiles^1^. Thus, testing with these lens types provides a direct assessment of the types currently available, and their different transmittance profiles may lead to differing effects on perceived speed. Note that BFL technologies are incorporated into spectacles without clear evidence of their actual benefits or potential effect on vision. The significance of this research is that raises the possibility that the selective filtering of blue light by BFLs may cause unintended perceptual changes in perception, particularly perceived speed which is essential to motion guided behaviours such as driving and judging time to contact.

## Methods

### Participants

Twenty participants between 18 and 35 years of age who had normal or corrected visual acuity took part in the study. The participants’ monocular and binocular visual acuity was screened with a Snellen chart for visual acuity of 6/6 or better. Those with significant eye conditions, amblyopia, strabismus, cataract, or corneal disease were excluded. Participants also undertook congenital colour vision testing with the Ishihara Test Book 24 Plate abridged edition, and the data from participants who did not pass were excluded. As the present study is the first to investigate the effect of BFLs on perceived speed, the goal of the research was not to establish population norms but was designed to be exploratory in nature and aimed to characterise speed perception changes due to blue light filtering lenses comprehensively in a small number of observers. As this is the first study to investigate this relationship, it was designed as an exploratory study^31^ that psychophysically established whether wearing BFLs affected image speed. This study adhered to the Tenets of the Declaration of Helsinki. Ethics approval for this study was granted by the University of New South Wales Australia Human Research Ethics Advisory Panel (reference number: HC190335). All participants signed an informed consent form before participating in the study.

### Stimuli

The stimuli, generated using custom-written software in MATLAB version 9, were of an achromatic or chromatic ring of 8 moving Gabor patches (Fig. 2). The Gabor patches were equally spaced 45° apart on the circumference of the circle of radius 4°. The equation for the Gabor patches was:

1 $$ G\left( {x, y} \right)  = e {-}\left( {x2 + y2} \right)/2s2 \times  \cos \left[ {2p \times  \left( {\cos q  \times x \, + \sin q \times y} \right)/p + f} \right], $$

where the orientation is determined by* q* and the relative phase of the element is *f*. This led the Gabor patches to be created from an oriented sinusoid as well as a circular Gaussian. Of the modulating sinusoid, the period or spatial frequency *p* was 1 cycle/degree. The approximate size, *s*, of the element was at 0.75° at half of its height and at the full width of the visual angle. All stimuli had a Weber contrast of 0.5 (peak to background), where the background was mid-grey of 9 cd/m^2^ in luminance. The position of the Gabor patches was fixed, and the movement was induced by the systematic change in the sinusoid phase across movie frames.

Different speeds were produced by increasing or decreasing the rate of phase change across 8 movie frames, with each frame shown for 33 ms. The total duration of each stimulus presentation was 264 ms. The four stimuli were identical except for colour. Each experiment involved either an achromatic, blue (CIE 1931 xy: 0.15, 0.06), green (CIE 1931 xy: 0.3, 0.6) and red (CIE 1931 xy: 0.64, 0.35) stimuli. Chromatic stimuli corresponding to the maximum output of the sRGB monitor and have dominant wavelengths overlapping with the transmittance profiles of the BFLs used in the present study. Figure 1C shows the spectral power distribution for the achromatic, red, green and blue stimuli used in the present study. The spectral distribution of the stimuli was quantified using a Cary 5000 UV–Vis–NIR spectrophotometer (Model: EL04043683) in which the spectral irradiance was measured across a range of wavelengths (300–700 nms).

The actual luminance contrast of all gratings was matched (0.5) to ensure that any potential change in contrast can be attributed to the BFLs. Additionally, chromatic gratings were additionally modulated in colour from the background grey.

As mentioned, in the introduction, because of their optical design, BFLs selectively block blue light, but do not impede the transmission of light at longer wavelengths. To provide an estimate of this effect, we measured the luminance of the 4 targets (blue, red, green and achromatic) and the gray background through each of the three BFLs using a Konica CS Minolta CS-100A luminance and colour photometer (see Table 1, for values in cd/m^2^, and in text below for Weber values). These values were then used to calculate Weber contrast values (Eq. (2)) and used to determine the degree to which stimulus contrast was actually reduced by BFLs compared to the clear control-lense.

2 $$ {\text{C}}_{{{\text{Weber}}}} = \, \left( {{\text{L}}_{{{\text{Stim}}\_{\text{Max}}}} - {\text{L}}_{{{\text{Bg}}}} } \right)/\left( {{\text{L}}_{{{\text{Bg}}}} } \right), $$

where L_Stim_Max_ is the maximum luminance of the stimulus, and L_Bg_ is the luminance of the background.

**Table 1:** Luminance measurements (in cd/m^2^) of the background and stimuli when taken through the three BFL lenses.

BFL	Stimulus type				
	Background	Blue	Red	Green	Achromatic
Blu-OLP	8.2	10.8	11.3	12.2	11.5
Crizal Prevencia	8.6	11.8	12.4	12.7	12.2
Blue Guardian	8.8	12.4	13.1	13	12.5

We find that the BFLs reduced stimulus contrast, but this was dependent on the stimulus colour and BFL. The Blu-OLP lenses reduced the contrast of blue, green, red and achromatic stimuli by the most consistent with its transmittance properties. It reduced the contrast of blue, green, red and achromatic stimuli (from their actual contrast of 0.5) to approximately 0.32, 0.38, 0.44 and 0.4. For the same colours the Crizal Prevencia stimulus contrasts were reduced to 0.38 (blue), 0.44 (green), 0.48 (red) and 0.42 (achromatic). For the Blue Guardian contrast reductions to 0.42 (blue), 0.49 (green), 0.48 (red) and 0.43 (achromatic) were recorded. From these measurements, BFLs selectively reduce the contrast of a blue stimulus more than the other stimuli. Interestingly, the contrast of the achromatic stimulus was notably affected by BFLs. This perhaps can be explained by the fact that the spectral distribution of the achromatic stimulus (dotted line in Fig. 1C) is not uniform across all wavelengths, but peaks at shorter wavelengths. Thus, the achromatic stimulus unintentionally comprised of more blue light. In hindsight this was perhaps related to the output capabilities of the monitor. This peak was not evident for the spectral distribution of the background which was set at a much lower luminance and was relatively uniform across different wavelengths.

Finally, regarding the stimuli used in the present study, participants judged the speed of computer-generated stimuli presented on an LCD monitor. This allowed us to completely control the speed, luminance, and colour profile of the stimulus, but at the expense of ecological validity because their spectra are somewhat curtailed (see Fig. 1C) and dissimilar to natural and or real-world conditions (see “General discussion” section).

Participants were seated in front of a True3-di 24-inch stereo-monitor which was level with their eyes, and dichoptically viewed the screen at a distance of 110 cm in a dark room. The stereo-monitor had separate LCD panels (60 Hz, resolution: 1920 × 1200) and a polarising plate separately presented the images to the eyes. The participant wore polarised lenses which ensured that one image was presented to one eye and another image to another eye in a dichoptic manner.

Participants wore custom-made fit-over goggles with removable lenses that were lightproof and eliminated reflections from the back of the lenses, so the only light assessed was the light transmitted from the monitor and travelling through the front of the lenses. One of the three BFLs was chosen in a randomised order and placed in the goggles in front of the right eye. The left eye always viewed the monitor through the clear control lens. A total of 4 lenses were used for each participant. If the participant usually wore spectacles, then the experimental lenses and polarised lenses were in front of their own spectacles. All lenses and the participant’s glasses were cleaned throughout the study.

This was a single-blinded study, and the participants were neither aware of which lenses they wore nor which eye was viewing through the control lens. The participants undertook the experiment in low photopic conditions. The stereo-monitor’s background luminance was 9 cd/m^2^ as measured with the Konica CS Minolta CS-100A.

The seated participants were allowed 5 min to adjust to wearing the goggles with the BFL and clear control lens. Participants were required to fixate on a target in the middle of the screen, and two stimuli, the reference and test stimuli, were presented centrally on the monitor screen in succession with an interstimulus interval of 500 ms in which the screen was blank and set to grey at the background luminance. A two-interval forced-choice design in conjunction with the Method of Constant Stimuli was used to measure perceived speed. The participant was required to judge which interval contained a faster-moving stimulus by pressing keys on a keyboard. After they had inputted their response, the next two interval stimuli were presented after a brief pause of 200 ms. The stereo-monitor and the polarised lenses allowed for dichoptic viewing, with each of the two stimuli presented exclusively to a different eye. The left eye always viewed the test stimuli through a BFL, and the right eye viewed the reference stimulus through the clear control lens. The presentation order of the test and reference stimuli was randomised from trial to trial. The Gabor elements in the test stimuli moved at one of 7 speeds: 1.5, 2, 2.5, 3, 3.5, 4, 4.5°/s, while the reference stimulus’ Gabor elements always moved at 2.75°/s. Each of the test speeds was presented at least 20 times in a randomised order. These procedures were repeated for 12 different stimulus conditions in which perceived speed was measured for 3 different BFLs and 4 stimulus colours.

## Results and discussion

**Figure 3:**
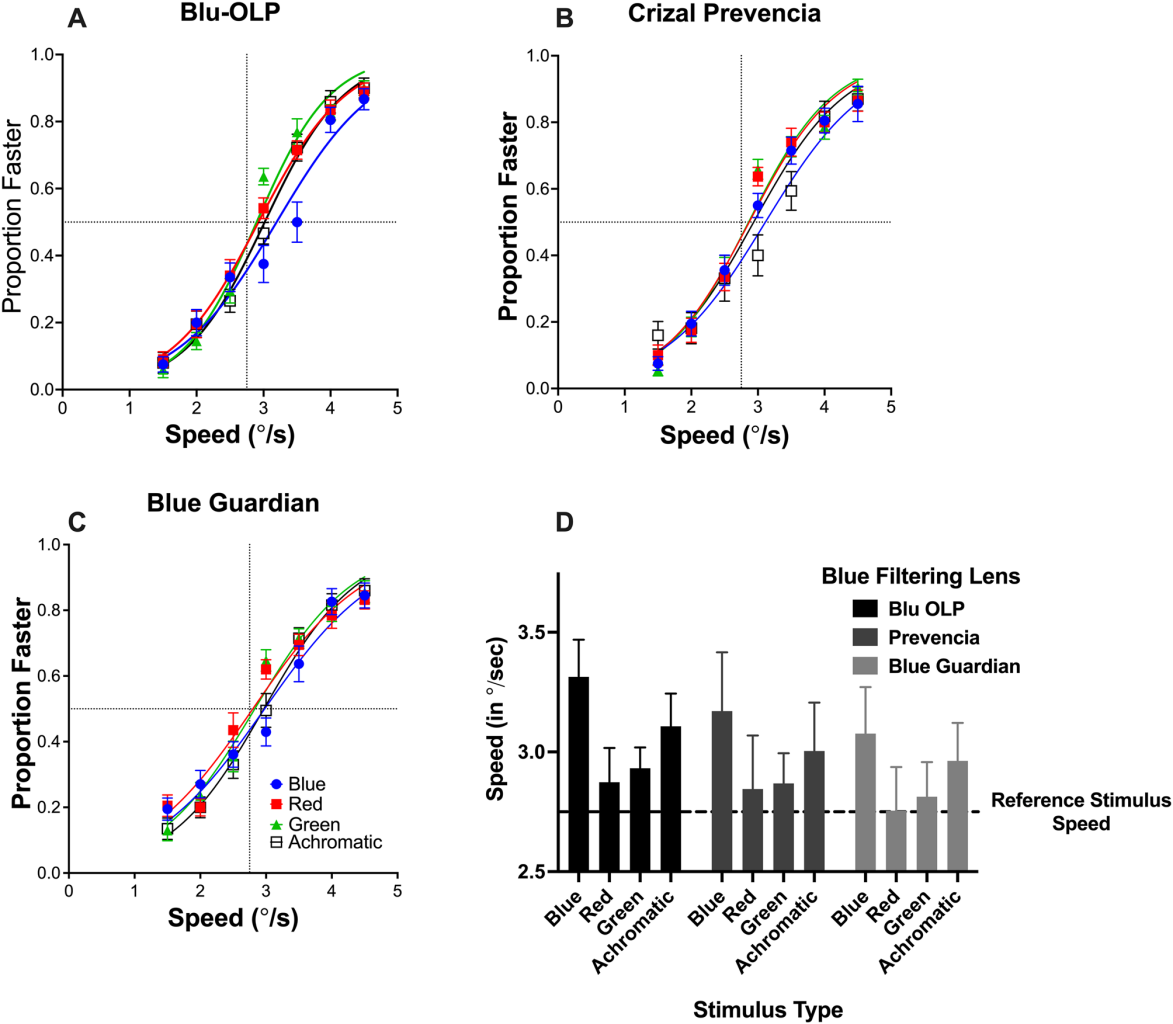
The average proportion of times the test stimulus was judged to be moving faster than the reference stimulus is plotted as a function of the test stimulus speed in (**A**–**C**), for different coloured stimuli (different colours and symbols). Best fit logistic function fit to data are also shown (solid lines). Error bars represent 95% confidence intervals. In (**D**), the PSE derived from each psychometric function is plotted each BFL and stimulus type. Dotted line represents the speed of the reference stimulus (2.75°/s).

Results for different participants and for the 3 BFLs and 4 stimulus colours (red, green, blue and achromatic) were collated, with all data passing the Shapiro–Wilk normality test. The proportion of times in which the test stimulus (viewed through a BFL) was judged to be moving faster than the reference stimulus was plotted as a function of the test stimulus speed. Logistic functions were then fitted to these data using GraphPad Prism (version 6). To provide an initial indication of any changes in perceived speed, the data from all 20 participants were collated and curve fits were conducted on the combined data. These data and fits are shown in Fig. 3A–C collated for different BFLs and stimulus colours. Fitted curves provided an estimate of the point of subject equality (PSE) which estimated the speed required to match the perceived speed of the reference stimulus which was viewed through the clear lens. Evident in this figure is that psychometric functions, particularly for blue and achromatic targets, were shifted to the right, which indicated that BFLs reduced the speed of the test stimulus, and accordingly, the test stimulus had to physically move faster to match the reference stimulus speed. Note that achromatic targets were also reduced in perceived speed. As mentioned previously, this is likely due to the fact that the spectral distribution (see Fig. 1C, dotted line) largely comprised of blue light, which would have been attenuated by BFLs. Additionally as reported in the methods section the actual contrast of the achromatic stimulus was lower than for red and green targets. Importantly, these results suggest that it is contrast and not the actual colour of the stimulus that is important to the perception of speed.

Additionally the extent of this effect was dependent on the type of BFL. In general, the data were well fitted by the logistic function as indicated by r^2^ > 0.72 and the 95% confidence limits (profile likelihood) close to the estimated PSE despite that there were some inter participant variability in the data (see Fig. 3). For Blu-OLP r^2^ goodness of fit values for blue, green, red stimuli were 0.78 (PSE: 3.195 (95% CI 3.1–3.3), 0.80 (PSE: 2.9 (95% CI 2.8–3.0), 0.87 (PSE: 2.92 (95% CI 2.8–3.1) and 0.81 (PSE: 3.0 (95% CI 2.9–3.1) respectively. Similar fit values were observed for Crizal Prevencia lens for blue (0.72, PSE: 3.1 (95% CI 3–3.2)), green (0.82, PSE: 2.86 (95% CI 2.8–3.0)), red (0.76, PSE: 2.8 (95% CI 2.7–2.9)) and achromatic (0.73, PSE: 2.92 (95% CI 2.8–3.0)). While for the Blue Guardian goodness of fit values for the different stimuli were 0.74 (blue, PSE: 3.0 (95% CI 2.9–3.1)), 0.73 (green, PSE: 2.8 (95% CI 2.7–2.9)), 0.79 (red, PSE: 2.8 (95% CI 2.7–3.9)) and 0.73 (achromatic, PSE: 3.0 (95% CI 2.9–3.1)).

To statistically evaluate whether perceived speed is affected by BFLs, we reanalysed our data and curve fitted the data from individual participants to derive an estimate of the PSE which we collated for the 4 different stimuli and 3 BFLs. Figure 3D plots the average PSE for different BFLs and stimulus types as bar graphs. Error bars signify 95% confidence intervals relative to the mean. The dotted line represents the speed of the reference stimulus. One sample t-tests were conducted to determine if the change in PSE speed for different BFLs and stimulus types were significantly different from reference stimulus speed of 2.75°/s. The PSEs values the Blu-OLP lens, was significantly different for the blue (mean: 3.314°/s, (t (19) = 7.592, p < 0.0001), achromatic stimuli mean (mean: 3.107°/s, (t (19) = 5.458, p < 0.0001), and green (mean: 2.932°/s, (t (19) = 4.389, p < 0.0003) but not for the red stimulus (mean: 2.873°/s, (t (19) = 1.805, p = 0.0869). Importantly, viewing blue, achromatic and green coloured stimuli through the Blu-OLP reduced their perceived speed by approximately 20, 13 and 6.6% respectively. For the Crizal Prevencia BFL, the PSE speed of blue (mean: 3.107°/s, (t (19) = 3.575, p < 0.002) and achromatic stimuli (mean: 3.004°/s, (t (19) = 2.626, p < 0.0166) were significantly lower than the reference stimulus, but the PSE speed for red (mean: 2.845°/s, (t (19) = 0.889, p = 0.385) and green stimuli (mean: 2.869°/s, (t (19) = 1.966, p = 0.0641) were not significantly different. Thus, the perceived speed of blue and achromatic stimuli was reduced by this BFL by approximately 15 and 9% respectively. A similar finding was observed for the Blue Guardian in which the PSE speeds for blue (mean: 3.077°/s, (t (19) = 3.524, p < 0.0023) and achromatic test stimuli (mean: 2.962°/s, (t (19) = 2.801, p < 0.0114) was significantly reduced by approximately 11 to 8% respectively, but no significant reduction in speed was observed for red (mean: 2.755°/s, (t (19) = 0.05633, p = 0.374) and green stimuli (mean: 2.813°/s, (t (19) = 0.910, p = 0.374).

In summary, the perceived speed of blue and achromatic stimuli was significantly impacted by viewing them through BFLs, but red stimuli were unaffected regardless of the BFL type. The perceived speed of a green stimulus was only affected by the Blu-OLP lens. These findings can be accounted for by the individual transmission properties of each BFL and the colour spectra of the stimulus (see Fig. 1B,C) and the degree to which BFLs reduce the contrast of the stimulus (as reported in the Methods section). All BFLs effectively attenuated short wavelengths comprising the blue and achromatic stimuli and hence the perceived speed of these stimuli was reduced. Note that the achromatic stimulus comprised of light, while broadly distributed, peaked at short wavelengths. This may account for the fact that BFLs significantly impacted the perceived speed of this stimulus. All BFLs did not selectively attenuate longer wavelengths, and hence the speed of the red stimulus remained unaffected. The green stimulus was only affected by the Blu-OLP because the transmission profile of this lens extended towards medium wavelengths.

**Figure 4:**
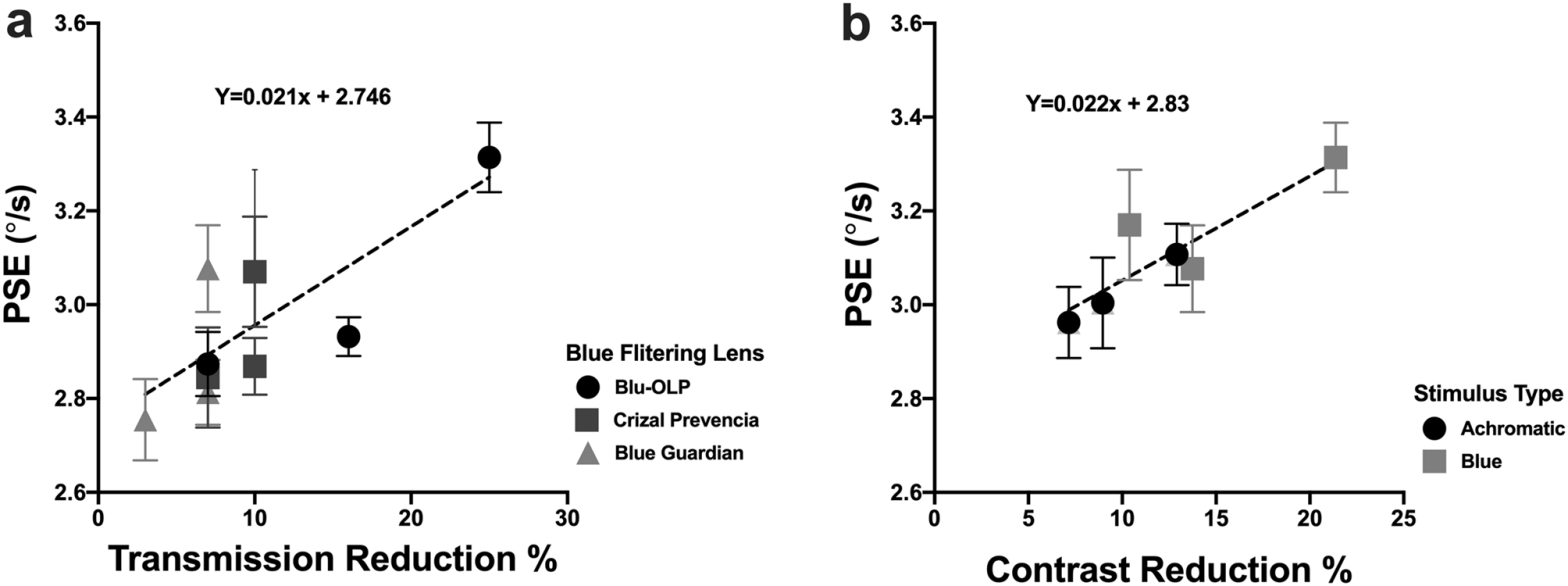
relating PSE change with the transmission (**A**) and contrast (**B**) reduction for different BFLs and coloured stimuli.

In Fig. 4A, a summary of the relationship between the transmission percentage reduction for the different BFLs at the peak dominant wavelength for each coloured stimulus (red, green and blue, derived from Fig. 1C) is plotted as a function of PSE speed. Linear regression analysis showed that that there is a significant correlation (Pearson R = 0.7435) between an increase in light transmission reduction and (slope: 0.0210 ± 0.007, F (1,7) = 8.652, p = 0.0217) increase in PSE speed. This indicated that BFLs, which filtered more light at a short wavelength was associated with a larger reduction in perceived speed, such that the test stimulus had to physically move faster (hence a faster PSE speed) to match the reference stimulus, which was viewed through a clear lens.

Finally, a two-way repeated-measures ANOVA was conducted to compare whether and how the type of BFL (factor 1: 3 levels) and stimulus colour (factor 2: 4 levels) significantly influenced perceived speed. Particularly, whether the extent to which perceived speed was reduced by BFLs was dependent on the type and colour of the stimulus. This analysis revealed a main effect of both colour (F (3,228) = 11.96, p < 0.0001) and BFL (F (2, 228) = 3.56, p = 0.0301), but there was no significant interaction effect (F (6,228) = 0.16, p = 0.9879) which indicated that the change in PSE for different stimulus colours was the same for the three BFLs. As shown in Fig. 3, in particular, the Blu-OLP lens and blue coloured stimuli (regardless of BFL type) is associated with greater changes in perceived speed.

### Relating perceived speed and luminance contrast

The present study reported that perceived speed is reduced by BFLs and this was associated with the degree to which BFLs attenuated light at particular wavelengths and thus as already demonstrated in the present study reducing the contrast of stimuli comprising of shorter wavelengths more than longer wavelengths. However, previous reports relate perceived speed with a reduction in perceived luminance contrast as both are visual perceptions. However, as shown in Fig. 4a, is it is expected that reduced light transmission (a property of BFLs) would lead to a reduction in stimulus contrast. For completeness, we conducted another study that quantified the degree to which BFLs reduced luminance contrast and related this to perceived speed.

The same observers as in the main study underwent additional testing in which their contrast perception was measured using the same BFLs to determine the extent to which they reduced perceived luminance contrast. The same configuration was used as in the main experiment, but stimuli were static, and the Weber luminance contrast of the stimulus was systematically varied from 0.3 to 0.7 at 0.1 steps (5 contrast levels).

Method of Constant Stimuli incorporating a two-interval forced-choice procedure was utilised in which a test stimulus was presented at one of the five contrast levels (at least 10 times), and the other interval contained a reference stimulus whose contrast was always fixed at 0.5. The task of the observer was to indicate the interval containing the higher contrast stimulus. This procedure was repeated with all three BFLs, but only with blue and achromatic stimuli were tested as these stimuli resulted in the largest reduction in perceived speed when viewed through BFLs.

**Figure 5:**
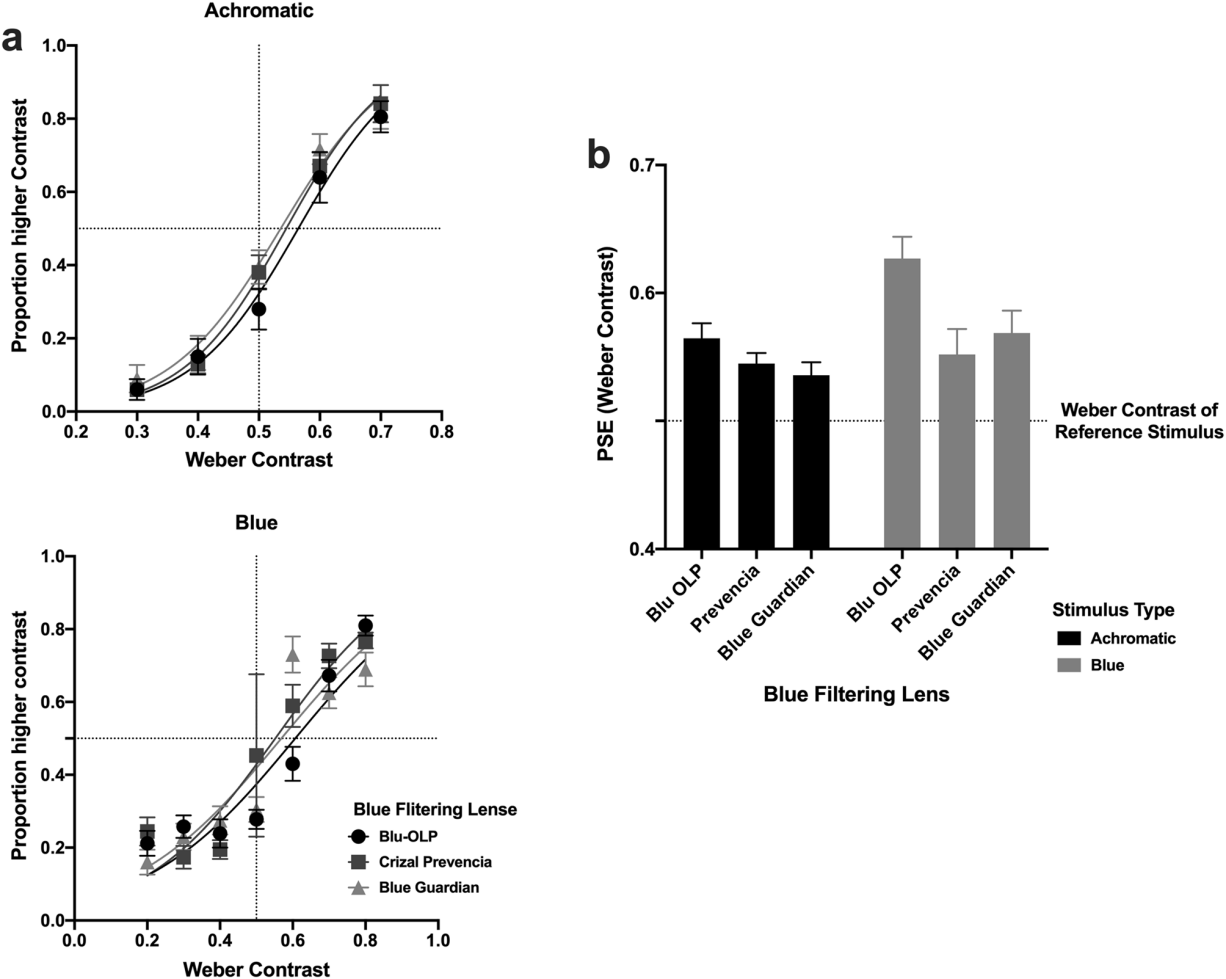
The proportion of times in which the test stimulus was judged to be higher in contrast than the reference stimulus is plotted as a function of the test stimulus contrast in (**a**). In (**b**), the PSE of the best fit psychometric function is plotted for each BFL and stimulus colour. Error bars represent 1 standard error of the mean.

Similar to the main experiment, for each BFL and stimulus colour the proportion of times in which the test stimulus was judged to be higher in contrast than the reference stimulus was plotted against the actual contrast of the test stimulus and logistic functions were fitted to these data to estimate the PSE, which in this case indicated the actual contrast of the test stimulus to match the reference stimulus. These psychometric functions and their corresponding PSE are shown in Fig. 5a,b for each BFL type and stimulus. These results showed that the image contrast of both blue and achromatic test stimuli, when viewed through the BFLs, was significantly reduced as the test stimulus had to be higher to match the contrast of the reference stimulus. All psychometric functions were shifted to the right, and PSEs were significantly higher than the reference contrast of 0.5 (one-sample t-test ps < 0.001), by approximately 5–20% but this was dependent on the BFL and stimulus colour. Indeed, a two way ANOVA confirmed that there was a main effect of colour (F (1,114) = 8.097, p = 0.0053) and BFL (F (2,114) = 6.399, p = 0.0023), but there was no significant interaction effect (F (2,114) = 1.764, p = 0.1760). Interestingly, as we have already observed the perceived contrast of the achromatic stimulus were affected by BFLs, and this we attribute to a peak in the spectral distribution of this stimulus at short-wavlengths.

In Fig. 4b, we compared and associated the effect of BFLs on reducing perceived speed and contrast regardless of the stimulus colour and BFL type. Linear regression analysis showed a significant positive relationship (Pearson, r = 0.8869, p = 0.0185) between these two visual behaviours such that increasing contrast reduction was associated with larger reductions in perceived speed. These findings implicate that image contrast is a major determining factor (r^2^ = 0.786) in perceiving speed consistent with previous reports^15–26^. Additionally, as noted previously, in the present we have measured the degree to which actual stimulus contrast is reduced by BFLs for the blue and achromatic stimuli (see “Methods” section). We find that the actual reduction in contrast is strongly associated with both perceived speed (Pearson r = 0.9552, p = 0.003) and perceived contrast (Pearson r = 0.9208, p = 0.0092).

## General discussion

Blue light filtering lenses are designed to reduce blue light transmission, which affects visual perception, which has been previously demonstrated to decrease perceived speed^15–26^. This present study demonstrates that using computer-generated stimuli in which their luminance and colour profiles can be specified, BFLs reduce perceived speed, but this was dependent on the BFL and stimulus colour. In particular, the perceived speed of blue and achromatic targets demonstrated the largest change in perceived speed, and the Blu-OLP lenses were the most effective in reducing perceived speed. These findings are expected since the Blu-OLP lens was the most effective in attenuating light transmission over a larger range of wavelengths compared to the other two brands (see Fig. 1b). All BFLs were effective in attenuating short wavelengths which accordingly impacted the contrast of blue and achromatic stimuli, which lead to a reduction in perceived speed. The perceived speed of red and green stimuli was largely unaffected, possibly because BFLs did not effectively reduce light transmission at medium and high wavelengths, and therefore their contrast remained unaltered.

While newer generation BFLs have been designed to limit exposure to harmful blue light, which our previous work^1,32^ has shown that they are effective in doing so, their use may have unintended consequences. In addition to changes in colour perception^1^ and reduced contrast^33^ due to BFL wear, our present study demonstrates that BFLs affects motion perception, particularly in reducing the perceived speed of objects. Where perhaps the implications of our finding are most applicable is driving behaviour. The work of Snowden et al.^27^ has shown that drivers speed up during foggy conditions, which coincides with a reduction in overall stimulus contrast. Our study implicates a similar effect when wearing BFLs, and importantly, their use during foggy conditions may present danger to the driver.

Our findings that BFLs may reduce perceived speed by approximately 6–20% would obviously impact the ability of the observer to estimate the time to contact of objects. In the absence of any other available cues (e.g., speedometer or landmarks) and if the observer is only reliant on object contrast to judge perceived speed, an expectation may be that a driver may drive faster. Importantly this may mean that there is less braking time to avoid a potential obstacle or stopping in time for traffic lights or obstacles such as pedestrians, animals and roadwork.

Driving behaviour is highly dependent on other visual cues (e.g., landmarks and signs) and actual indicators of car speed. However, the reduction in perceived speed noted in the present study was dependent on the object colour, and we utilised artificial computer-generated objects with restricted colour spectra, unlike real-world objects that may have broad colour spectra. Thus, the testing conditions utilised in the present study was ideal and highly controlled and are not immediately applicable to real-world driving conditions in which a number of cues and information is available to the driver to check the speed of travel. Future studies may which to examine speed perception under real-world conditions, but this beyond the scope of the present study, which sought to initially confirm whether and how BFLs affect speed perception.

Additionally, the overall luminance of stimulus would likely to be a major contributing factor to the potential effect of BFLs on perceived speed. Note that we used comparatively low contrast stimuli under low phototopic conditions, and this is likely to lead to a larger effect as previous studies have reported greater reductions in perceived speed with low contrast objects^18^. It is not unreasonable to expect that BFLs would have a negligible effect under daytime driving conditions but may affect driving performance during night-time driving where overall luminance is considerably reduced. However, it is important to note that BFLs may actually increase perceived speed during night-time driving because BFLs may enhance object contrast by attenuating background luminance. While our preliminary results suggest that BFLs may reduce perceived speed, the impact of this warrants further examination in real-world conditions to establish whether and the degree to which BFLs are a safety risk to driving behaviour.

## Conclusions

Commercially available BFLs, which can be a feature of spectacles, have been shown to effectively attenuate blue light exposure, particularly at short wavelengths known to influence the circadian rhythm and cone function^1,34^. The present study demonstrated that this attenuation of blue light may reduce image contrast which can lead to a significant reduction of perceived speed under controlled stimulus conditions. This outcome highlights that selectively blocking light may have consequences for visual function and object perception and, ultimately, how visual information is utilised for visually guided behaviour.
